# Antibacterial Bionanocomposites Based on Drug-Templated Bifunctional Mesoporous Silica Nanocontainers

**DOI:** 10.3390/pharmaceutics15122675

**Published:** 2023-11-25

**Authors:** Elena M. Shishmakova, Anastasia V. Ivchenko, Anastasia V. Bolshakova, Maxim S. Staltsov, Ekaterina K. Urodkova, Natalia E. Grammatikova, Victor M. Rudoy, Olga V. Dement’eva

**Affiliations:** 1 Frumkin Institute of Physical Chemistry and Electrochemistry, Russian Academy of Sciences, 119071 Moscow, Russia; anastasia.ivchenko22@gmail.com (A.V.I.); nastya@polly.phys.msu.ru (A.V.B.); urodkovakatja@mail.ru (E.K.U.); redmoun@mail.ru (V.M.R.); 2 Department of Chemistry, Moscow State University, 119992 Moscow, Russia; 3 Division of Nuclear Physics and Technologies, National Research Nuclear University MEPHI, 115409 Moscow, Russia; m.staltsov@gmail.com; 4 Gause Institute of New Antibiotics, 119021 Moscow, Russia; ngrammatikova@yandex.ru

**Keywords:** mesoporous silica nanoparticles, drug-templated synthesis, solubilization, hydrophobic drug encapsulation, alginate hydrogels, antibacterial materials

## Abstract

The creation of antibacterial nanocomposites that provide prolonged release of encapsulated drugs is of great interest for various fields of medicine (dentistry, tissue regeneration, etc.). This article demonstrates the possibility of creating such nanocomposites based on sodium alginate and drug-templated mesoporous silica nanocontainers (MSNs) loaded with two bioactive substances. Herein, we thoroughly study all stages of the process, starting with the synthesis of MSNs using antiseptic micelles containing the hydrophobic drug quercetin and ending with assessing the activity of the resulting composites against various microorganisms. The main emphasis is on studying the quercetin solubilization in antiseptic micelles as well as establishing the relationship between the conditions of MSN synthesis and micelle morphology and capacity. The effect of medium pH on the release rate of encapsulated drugs is also evaluated. It was shown that the MSNs contained large amounts of encapsulated drugs and that the rate of drug unloading depended on the medium pH. The incorporation of such MSNs into the alginate matrix allowed for a prolonged release of the drugs.

## 1. Introduction

The encapsulation of different (primarily hydrophobic) functional compounds and their controlled release into the environment is a highly relevant problem in biomedicine, pharmaceuticals, material protection, and other fields [1,2,3,4]. In this regard, mesoporous silica nanoparticles (MSNs) are of considerable interest. They have attractive properties, such as a high specific surface area, an ordered pore system, relatively low toxicity, and the ability to degrade in an aqueous environment over time [4,5]. As a result, such MSNs are widely used as drug delivery vehicles [1,4] as well as in the creation of various types of coatings. It has been shown, for example (see [2,5,6,7,8,9] and the references therein), that the incorporation of MSNs loaded with corrosion inhibitors into polymer coatings can significantly increase the effectiveness of their protective effect. In our opinion, MSNs are also interesting when creating nanocomposites for biomedical applications (e.g., wound dressings or dental materials) [5,10,11,12]. The main method of obtaining MSNs is sol–gel synthesis using surfactant micelles as a template. The creation of delivery systems using “inert” surfactants (i.e., performing only a structure-directing function) requires the implementation of the following multi-stage algorithm [5,10,11,12]:

- The synthesis of MSNs;
- Template removal by calcination or chemical etching;
- The modification of the inner and/or outer surface with a functional compound having an affinity for the loaded drug;
- Drug loading;
- An additional modification of the MSN surface to ensure that the drug release occurs “at the right time in the right place”.

The complexity of the algorithm, which involves the use of several modifiers, is a serious drawback that makes it difficult to scale up the process. Another inevitable drawback of the traditional algorithm is the relatively low capacity of the synthesized MSNs for the target substance, which usually does not exceed 10–20 wt% [5]. 

**Scheme 1:**
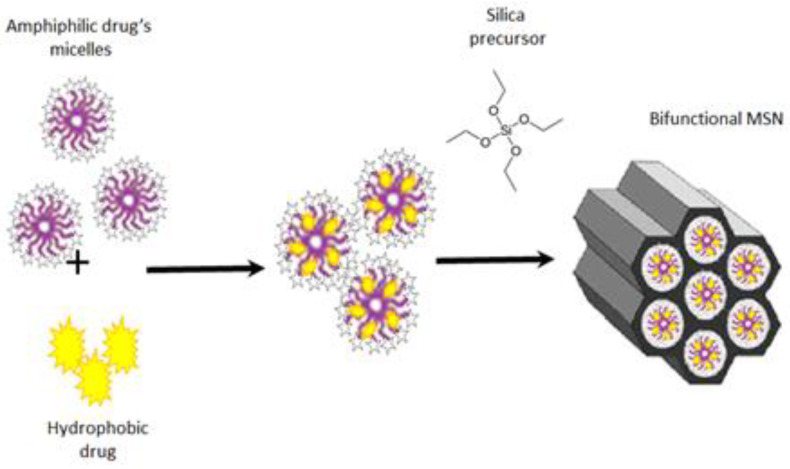
The common scheme of synthesis of MSNs, which are loaded with two drugs.

These shortcomings have led to the emergence and fairly rapid development of an alternative approach [5,8,9,13,14,15,16]. It is based on the use of an amphiphilic *functional* compound (e.g., a drug or corrosion inhibitor) as a templating agent. It allows one to combine the stages of particle synthesis and to load not only one but several target compounds [5,8,9,14,15,16]. This possibility exists due to the ability of hydrophobic compounds to be solubilized in surfactant micelles. It is known [16,17,18,19] that micellar solubilization can significantly enhance the water solubility (i.e., bioavailability) of hydrophobic drugs. Moreover, surfactant micelles containing a hydrophobic solubilizate can serve as effective hybrid templates in the synthesis of MSNs [15,16]. Thus, in the course of our experiments [16], this approach was successfully used to obtain MSNs and to simultaneously load them with two drugs. One of the drugs is the micelle-forming antiseptic miramistin and the other is curcumin, a hydrophobic, biologically active compound with a wide spectrum of action [20]. The general scheme for the formation of such MSNs is given below (Scheme 1).

MSNs obtained using functional templates are characterized by a high capacity (up to 50 wt% or more). Moreover, they are sensitive to medium pH. It allows one to finely control the release rate of the encapsulated substance without any additional modifications to the MSNs’ surface [5,8,9,13,14,15,16]. In particular, it has been shown [8,9] that the introduction of such MSNs into paint coatings provides long-term corrosion protection of metal alloys due to the prolonged release of encapsulated compounds.

It can be expected that the same prolonged release will occur when drug-templated MSNs are combined with biocompatible polymers. Sodium alginate, which is a non-toxic, biodegradable polysaccharide, is of significant interest in this regard. Owing to a high swellability, sodium alginate films are widely used as scaffolds in different biomedical applications, especially wound dressings and tissue repair materials [21].

This work is a continuation of studies begun in [16]. Its main objective is evaluating the prospects of the creation of antibacterial nanocomposites based on sodium alginate and bifunctional MSNs. To verify the versatility of the proposed one-pot approach to the synthesis of such MSNs, another hydrophobic substance, quercetin, was used. To this end, quercetin solubilization in miramistin micelles and the subsequent formation of MSNs on such a hybrid template as well as the kinetics of templating drug release were thoroughly studied.

## 2. Materials and Methods

In this work, we used tetraethoxysilane (TEOS), quercetin, ammonium fluoride, 28 wt% aqueous ammonia solution, 1 N aqueous sodium hydroxide solution (all are ACS reagent grade, Sigma-Aldrich, Darmstadt, Germany), anhydrous ethanol (reagent grade, Component-Reaktive, Saint Petersburg, Russia), sodium alginate, calcium chloride, phosphate buffer (EKOlab, Elektrogorsk, Russia), 37% hydrochloric acid (reagent grade, Chimmed, Moskva, Russia). Miramistin was kindly provided by Infamed (Moscow, Russia).

Freshly distilled 96% ethanol and distilled water were used as solvents. 

All chemical glassware was treated with a chromic mixture and then repeatedly rinsed with distilled water. 

### 2.1. Quercetin Solubilization in Miramistin Micelles

The study of quercetin solubilization in antiseptic micelles was carried out according to the classical scheme described in [17]. A series of 4.0 mL aqueous micellar solutions of miramistin with concentrations of 1, 3, 6, and 9 mM was prepared. Then 5 mg (1.25 mg/mL) of quercetin was added to each solution. The solutions were stirred in the dark at 25 °C. After specified time intervals (1–144 h), samples were taken from the solutions and centrifuged to separate the insoluble (i.e., non-solubilized) quercetin. The selected supernatants were diluted 100-fold with ethanol, and their absorption spectra were recorded. The value of quercetin solubilization was determined using a calibration curve—the concentration dependence of the optical density of its ethanolic solutions at a wavelength of 375 nm (Figure S1a).

An Evolution 300 UV–Vis double-beam spectrophotometer (Thermo Electron Corporation, Waltham, MA, USA) was used to determine the amount of quercetin solubilized in the miramistin micelles. The same equipment was used to evaluate the encapsulation efficiency of the target compounds in MSNs and analyze the release rate of both compounds into a dispersion medium (including from alginate/MSN composites). Spectra were recorded in the wavelength range of 190–800 nm using quartz cuvettes with an optical path length of 1 cm, and a similar cuvette was placed with the same solvent in the reference beam.

### 2.2. Synthesis of MSNs on a Hybrid Template 

The synthesis of MSNs in an alkaline medium was carried out as follows: 1 mL of aqueous ammonia solution was added to 24 mL of 3 mM aqueous miramistin solution with solubilized quercetin followed by 1 mL of TEOS solution in absolute ethanol (15 vol%). Then the reaction system was intensively stirred for 24 h in the dark at 25 °C.

During the synthesis of MSNs in a neutral medium, 2 mL of NH_4_F solution (25 mg/mL) was added to 24 mL of 3 mM aqueous miramistin solution with solubilized quercetin. Then 1–2 mL of alcohol solution with TEOS (15–30 vol%) were added to the system 50 μL portions at a time at 5 min intervals and intensively stirred for 24 h in the dark at 25 °C. MSNs were synthesized in a similar way on «empty» (i.e., without quercetin) miramistin micelles. 

The obtained particles were precipitated from the colloidal solutions by centrifugation (45 min at 14,000 rpm) using a Universal 320R centrifuge (Hettich, Kirchlengern, Germany) and then redispersed in 96% ethanol or aqueous solutions with given pH values in an ultrasonic bath. 

The supernatant was separated and analyzed by electronic absorption spectroscopy. The amounts of quercetin and miramistin incorporated into MSNs were determined using the corresponding calibration curves (Figure S1a,b). The precipitate was redispersed in distilled water. The precipitation/redispersion procedure was repeated twice. Part of the resulting precipitate was dried in a vacuum oven at room temperature and studied using IR spectroscopy and thermogravimetric analysis (TGA). The remaining dispersion was used to analyze particle morphology by high-resolution transmission electron microscopy (HRTEM) and to carry out further experiments in order to study the kinetics of encapsulated drug release from MSNs and to create alginate/MSN composites.

### 2.3. Characterisation of MSNs

The size and porous structure of the MSNs were determined using Leo-912 AB Omega and Libra 120 HRTEM microscopes (both manufactured by K. Zeiss, Oberkochen, Germany) operating at accelerating voltages of 100 and 120 kV, respectively. The samples were prepared by holding a drop of aqueous dispersion on a formvar-coated copper grid for 1 min and then removing it with filter paper. 

The incorporation of the target compounds into the silica matrix of MSNs was qualitatively evaluated using a Nicolet 380 FTIR spectrometer (Thermo Electron Corporation, Waltham, MA, USA). The spectra were measured in diffuse reflectance mode in the wavenumber range of 400–4000 cm^−1^. 

The total amount of drugs encapsulated in the MSNs was determined by TGA. The experiments were carried out using a TGA Q500 instrument (TA Instruments, New Castle, DE, USA) in open platinum crucibles under argon flow in a temperature range of 25–600 °C at a heating rate of 10 °C/min. The samples were preliminarily dried for 24 h at room temperature in a VD 23 vacuum drying oven (Binder, Tuttlingen, Germany).

### 2.4. Preparation of Alginate Films and Creation of Alginate/MSN Composites

The films were obtained by casting from a pristine or MSN-containing aqueous solution of sodium alginate. Composites were created using MSNs synthesized in a neutral medium on empty or quercetin-containing miramistin micelles.

Aqueous solutions of sodium alginate (with a concentration of 40 mg/mL) were prepared by dissolving weighed portions of 0.4 g of the polymer in 10 mL of distilled water with continuous stirring for 3 h at 25 °C. To create alginate/MSN composites, an aqueous solution of sodium alginate was mixed with an aqueous dispersion of MSNs in a volume ratio of 1:1, stirred for 1 h, and kept under vacuum for several minutes to remove air bubbles. Films were cast from the resulting colloidal solution on glass or fluoroplastic substrates and dried at 25 °C for a day. The content of MSNs in composite films was ~34 wt%.

The crosslinking of the obtained films was carried out according to the scheme proposed earlier [22]. The film was placed in 10 mL of a water–alcohol (10–40 vol% ethanol) solution containing calcium chloride at a concentration of 1.5 wt%, kept for 1 h, washed with water, and dried at 25 °C to constant weight. 

The swelling of pristine alginate and composite films was evaluated by their weight change upon exposure to distilled water. (Note that the equilibrium swelling values were reached after 1 h.) 

The morphology of the alginate/MSN composites was studied by atomic force microscopy (AFM) in tapping mode. Force curves for such samples were also recorded using AFM. The experiments were carried out on an atomic force microscope NanoScope V Multimode (Bruker, Billerica, MA, USA). For such measurements, we used polysilicon cantilevers HA_FM from TipsNano (Tallinn, Estonia) with a large aspect ratio, a resonance frequency of 77 kHz, and a stiffness of 3.5 N/m. All force curve measurements were performed using the same probe on the same day. The image processing was performed using FemtoScan Online 4.3 software (Advanced Technologies Center, Moscow, Russia, www.nanoscopy.ru (accessed on 21 October 2023)) [23,24,25,26]. The force curves were analyzed using the NanoScope Analysis 2.0 software (Bruker, Billerica, MA, USA).

### 2.5. Kinetic Study of the Encapsulated Drug Release into the Aqueous *Medium* from the MSNs and Alginate/MSN Composites 

The kinetics of the encapsulated drug release from the MSNs into the aqueous dispersion medium was studied under static and quasi-dynamic conditions at 25 °C. The pH of the solutions was measured using an I-500 ionometer (Akvilon, Podolsk, Russia).

In the first case, MSNs were dispersed in water with the required pH value and kept for a specified time. Periodically, samples were taken from the system and centrifuged. The absorption spectra of the obtained supernatants were analyzed to determine the amounts of both compounds that passed into the dispersion medium using the corresponding calibration curves (Figure S1a,b). (To determine the content of quercetin, the supernatant liquid was preliminarily diluted with ethanol 10–100 times.)

In the second case, after specified time intervals, the aqueous dispersion medium was replaced with a fresh portion (with the same pH value) by precipitation and redispersion of particles. The content of drugs released from MSNs was determined similarly by analyzing the supernatant liquid taken after centrifugation of each sample.

To evaluate the kinetics of drug release from alginate/MSN composites, the cross-linked films obtained according to the scheme described in Section 2.4 were used. The study was carried out in static conditions. Composite films were immersed in neutral (pH = 7) or slightly acidic (pH = 5) phosphate buffer. After specified time intervals, samples were taken from the system and their absorption spectra were analyzed according to the scheme described above.

### 2.6. Determination of Antibacterial Activity of Alginate/MSN Composites

The activity of the obtained composites was evaluated for *Staphylococcus aureus* ATCC 29213 by the agar diffusion method. In the experiments, we used both pristine and cross-linked composite films obtained according to the above-mentioned scheme (see Section 2.4). MSN-free sodium alginate films were used as a control. 

To prepare the inoculum, the culture of *Staphylococcus aureus* ATCC 29213 was activated after cryostorage by inoculation on trypticase soy agar (FBIS SRC ABM, Russia) and incubated for 18–24 h at a temperature of 36 ± 1 °C. After incubation, individual morphologically homogeneous colonies were suspended in saline with a turbidity equivalent to 0.5 units according to the McFarland standard, which corresponds to a titer of 1.5 × 10^8^ CFU/mL. Turbidity was measured using a Den-1 densitometer (Biosan, Riga, Latvia). 

Nutrient agar for activity assessment was prepared by standard procedures based on the powder “Dry nutrient agar for the cultivation of microorganisms (GRM-Agar)” (FBIS SRC ABM, Moscow, Russia).

The inoculum was added to the melted and cooled agar medium to a final titer of about 5 × 10^6^ CFU/mL, poured into 10 mL Petri dishes with diameters of 90 mm, and left until the agar solidified.

Alginate/MSN films were placed on the surface of the infected agar medium and kept for 2 h at room temperature for uniform diffusion of the preparations. Films were incubated at 36 ± 1 °C for 18–24 h. The activity was determined using the diameter of the inhibition zones around the films and averaging the results of 4 measurements. Each experiment was carried out in triplicate. 

## 3. Results and Discussion

### 3.1. Quercetin Solubilization in Miramistin Micelles 

First of all, we emphasized that there are currently just a few works devoted to the synthesis of MSNs on a hybrid template, which are micelles of inert [8,27,28] or functional surfactant [15,16] with a hydrophobic compound solubilized within them. In the vast majority of these works, all components are introduced into the reaction system almost simultaneously. The authors of these works did not consider the features of solubilization (e.g., the rate of this process and the content and localization of the hydrophobic substance in micelles). The lack of such information may adversely affect not only the reproducibility of the synthesis of MSNs but also their capacity and porous structure.

**Figure 1:**
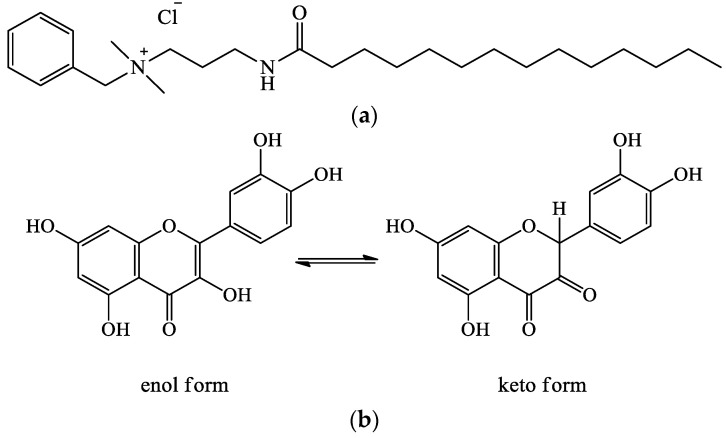
Chemical structures of miramistin (**a**) and quercetin keto-enol tautomeric forms (**b**).

With this in mind, our first step was to investigate the solubilization features of the hydrophobic drug. As already noted, we used the amphiphilic drug miramistin (cationic surfactant) with antibacterial and antifungal effects [29] as a templating agent in the synthesis of mesoporous particles (Figure 1a). Its critical micelle concentration (CMC) is approximately 1 mM [30]. In this work, we evaluated the possibility of using the miramistin micelles to increase the solubility of quercetin, a hydrophobic natural polyphenol with pronounced antimicrobial and wound-healing effects [31,32]. It exists in two tautomeric forms (Figure 1b). The absorption spectra of these compounds are shown in Figure S2.

**Figure 2:**
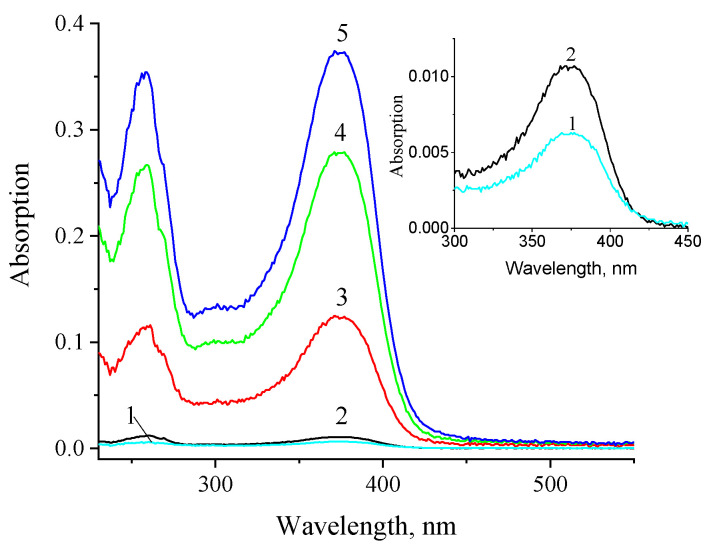
Absorption spectra of aqueous solution of quercetin (1) and miramistin micellar solutions with concentrations of 1 mM (2), 3 mM (3), 6 mM (4), and 9 mM (5) 1 h after the beginning of quercetin solubilization. Spectra 1 and 2 are also shown as inset.

According to our experiments, the quercetin solubilization in miramistin micelles proceeded rather quickly. Additionally, as the concentration of miramistin increased, the content of quercetin in the solution also increased. This is evidenced by an increase in the optical density of the solution at a wavelength of 375 nm (Figure 2). The equilibrium concentrations of quercetin in miramistin micellar solutions with concentrations of 1 and 9 mM were 70 µM and 2.1 mM, respectively, and were reached in about 1 h after the beginning of solubilization. These values significantly exceeded the concentration corresponding to the equilibrium solubility of quercetin in water, which, according to our estimate, is approximately 48 µM.

It should be noted that holding the micellar solution containing solubilizate for more than 1–3 h led to a gradual decrease in its optical density (Figure S3a,b). The magnitude of this effect increased with decreasing concentrations of miramistin. In this case, there was no noticeable change in the spectrum shape. This pattern may be associated with the quercetin oxidation by atmospheric oxygen. However, for a more detailed clarification of its reasons, more in-depth studies are needed that are beyond the scope of this work.

**Figure 3:**
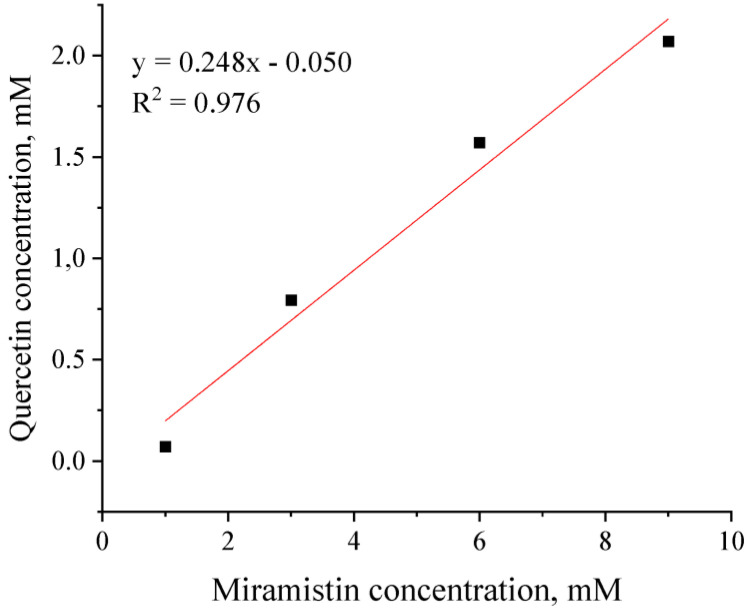
Solubilization isotherm of quercetin in aqueous micellar solutions of miramistin.

Figure 3 shows the isotherm of quercetin solubilization in miramistin micelles. It has a linear form (which indicates the invariance of micelle shape as the solubilization process proceeds) and is described by an equation of the form:

*y* = *ax* + *b*.

The parameter *a*, equal to 0.248, is the value of the solubilization capacity of the micelles, and *E* is the ratio of the number of moles of solubilizate (in our case, quercetin, *N*_q_) and solubilizer (*N*_surf_) in the micelle:

*E* = *N*_q_/*N*_surf_

The molar fractions of quercetin in the micelle (χ_m_) and in the aqueous phase (χ_w_) were calculated using the following formulas [16]:

χ_m_ = *N*_q_/(*N*_q_ + *N*_surf_) = *E*/(*E* + 1),

χ_w_ ≈ *S*_w_/*C*_w_ ≈ *S*_w_/55.43,

where *S*_w_ is the quercetin solubility in water, equal to 4.85 × 10^−5^ M, and *C*_w_ is the water concentration (55.43 M at 22 °C). The values of χ_m_ and χ_w_ are 0.20 and 8.75 × 10^−7^, respectively.

The distribution constant (*K*_d_) of the solubilizate between the micellar pseudophase and water and the standard Gibbs free energy of solubilization (Δ*G*°) were determined as

*K*_d_ = χ_m_/χ_w_,

Δ*G*° = −*RT*ln*K*_d_,

where *R* is the gas constant and *T* is the absolute temperature.

Our calculations showed that *K*_d_ is 2.29 × 10^5^ and that Δ*G*° is −30.27 kJ/mol. These values are quite close to those obtained earlier for the case of curcumin solubilization in miramistin micelles [16], which is quite natural, given the similar structure of quercetin and curcumin molecules.

The quercetin molecule contains two types of substituents—polar phenolic and keto groups and non-polar hydrocarbon radicals and aromatic systems. Therefore, the quercetin molecule can be located both in the hydrophobic hydrocarbon core and in the hydrophilic corona of the micelle as well as in its intermediate palisade layer. The similarity of the structures of the quercetin and curcumin molecules, the solubilization of which we studied earlier [16], suggests that quercetin is also localized precisely in the palisade layer of the miramistin micelles.

Thus, the data presented above indicate that the solubilization of quercetin in miramistin micelles does indeed significantly increase quercetin’s solubility in water (and therefore its bioavailability). Moreover, it can be expected that combining these two drugs should promote the healing of infected wounds. This may occur due to both their combined antibacterial action and the ability of quercetin to promote wound healing (for example, by modulation of macrophage polarization from M1 to M2 phenotypes [32]).

### 3.2. Synthesis of MSNs on a Hybrid Template and Creation of Alginate/MSN Composites

The next step was the synthesis of MSNs on a hybrid template, i.e., miramistin micelles containing solubilized quercetin. These experiments were carried out in alkaline and neutral media.

The addition of an alkaline NH_3_ · H_2_O catalyst to the miramistin micellar solution with solubilized quercetin changed its color from yellow to light green. In this case, the main absorption band of quercetin, with a maximum at 375 nm, was shifted to the short wavelength region up to about 320 nm (Figure S4). Simultaneously, a shoulder appeared in the absorption spectrum of the solution in the region of 350–400 nm. According to [33], the bands with maxima near 300 and 350 nm correspond to the ketone tautomer of quercetin in its molecular and anionic forms, respectively. Apparently, in our experiment, quercetin underwent a tautomeric transition from the enol to the ketone form; in this case, some of the molecules were deprotonated. The introduction of a silica precursor into such a system leads to its rapid clouding due to the formation of MSNs. 

**Figure 4:**
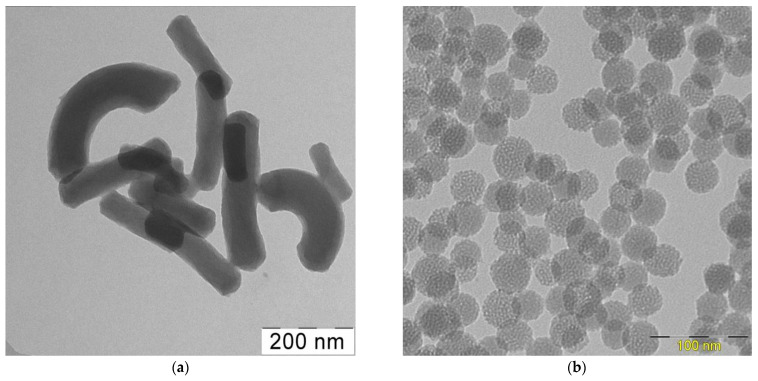
HRTEM images of the MSNs synthesized in alkaline (**a**) or neutral (**b**) medium.

The micrographs of the obtained particles are shown in Figure 4a. MSNs are rod-shaped particles with cylindrical, hexagonally ordered pores. The average diameter and length of the rods were 70 and 230 nm, respectively, i.e., their axial ratio was about 3. This picture is generally consistent with that observed previously when miramistin micelles containing curcumin were used as a template [16]. In the case of using quercetin, the addition of alkali also led to the deprotonation of its solubilized molecules. As a result, the charge of the miramistin head groups was partially neutralized, which means that their electrostatic repulsion decreased. This led to an increase in the curvature radius of the micelles, and their shape changed from spherical to rod-shaped. Moreover, this process can be facilitated by the interaction between the template and silicic acid oligomers during the formation of mesoporous particles [16]. 

Charge compensation did not occur in a neutral medium, and spherical MSNs with a narrow size distribution and a well-defined porous structure were formed during the synthesis process (Figure 4b). The morphology of such particles was almost identical to that observed for the MSNs obtained using “empty” (i.e., in the absence of solubilizate) miramistin micelles (Figure S5). At the same time, a twofold increase in the precursor content in the system led to an increase in the particle diameter from 35 to 65 nm (note that this leads to some increase in the relative content of miramistin in the particles).

In the IR spectra for both types of MSNs, in addition to the absorption bands near 1000 and 1250 cm^−1^, which are characteristic of Si–O–Si stretching vibrations, intense bands were recorded near 1650 and 2900 cm^−1^, which correspond to the stretching vibrations of C=O and C–H bonds in quercetin and miramistin molecules (Figure S6). This indirectly indicates the incorporation of sufficiently large amounts of both templating compounds in the silica matrix. Unfortunately, it is impossible to estimate the proportion of each templating compound due to the overlapping of their main absorption bands. 

The amount of quercetin encapsulated in the MSNs was determined from the absorption spectra of the supernatants taken after particle synthesis. The content of this drug in MSNs obtained in an alkaline medium turned out to be very low, about 1% of the weight of the silica matrix. This is due to the partial degradation of quercetin in an alkaline medium. Reducing the reaction system pH to 7 led to a significant (up to 10 wt%) increase in the quercetin content in the MSNs due to the greater stability of this flavonoid in a neutral medium. 

Miramistin determination from the absorption spectra of the supernatants was difficult due to the overlapping of the characteristic bands of both preparations near 260 nm (Figure S2). Therefore, TGA was used to estimate the amount of miramistin in the MSNs.

The main weight loss corresponding to the thermal degradation of the templating drugs was observed in the range of 150–350 °C and was approximately 55 wt% and 35 wt% (i.e., ≈1.2 and ≈0.47 g per 1 g SiO_2_) for the MSNs obtained in alkaline and neutral medium, respectively (Figure S7). Unfortunately, it was rather difficult to estimate the contribution of each of them due to the different nature of their degradation (insets in Figure S7). Thus, the degradation products of miramistin were mainly volatile compounds (in this case the weight loss is about 100%). In the case of quercetin, a relatively large number (30–40%) of non-volatile compounds were formed. Comparisons of the shapes of the TGA curves obtained from the pure drugs and the MSNs indicated that the main reason for the weight loss of both types of particles is the destruction of miramistin. Taking into account the results of absorption spectroscopy, it can be assumed that the amount of antiseptic was ≈54 wt% and ≈30 wt% for the MSNs synthesized in alkaline and neutral media, respectively. However, it should be emphasized that this estimate can be quite rough for the second type of MSNs containing a sufficiently large amount of quercetin. 

Significant differences in the miramistin content of different types of MSNs can be due to differences in their structural porosities and the variable nature of the interactions of miramistin cations with their pore walls. It is known, in particular, that as the pH of the medium decreases, the fraction of deprotonated silanols in the composition of silicic acid oligomers decreases, which weakens their electrostatic interaction with micellar template cations. This can lead to a decrease in the proportion of the antiseptic that is incorporated into the MSNs in a neutral medium. We also noted that the nature of the thermal degradation of the encapsulated compounds in particles obtained in different media is somewhat different. A detailed analysis of these differences is beyond the scope of this work. It should be emphasized that the thermal properties of many organic compounds, including drugs incorporated into MSNs, differ significantly from their corresponding bulk characteristics and are largely determined by the strength of the interaction of their molecules with the pore walls and pore size (see, for example, [34] and the references therein). 

The data presented above indicate that the use of a hybrid template, which consists of micelles of the antiseptic miramistin with solubilized quercetin, makes it possible to combine the stages of MSN synthesis and their loading with several functional compounds. In this case, the size, shape, and capacity of the resulting particles can be controlled by changing both the pH of the medium and the ratio of the components of the reaction system. 

The next step was to study the possibility of the incorporation the MSNs into the alginate matrix to create biologically active composites. Particles synthesized in a neutral medium on empty and quercetin-containing miramistin micelles were used as model fillers. The content of miramistin in these two types of MSNs was approximately the same.

**Figure 5:**
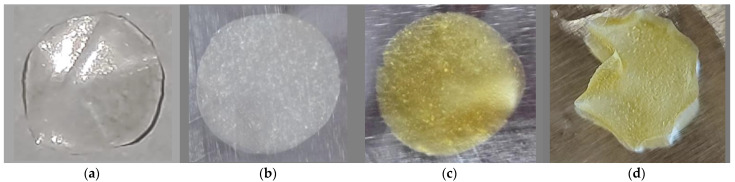
Photographs of sodium alginate films (**a**) and alginate/MSN composites with particles containing miramistin (**b**) or its combination with quercetin (**c**,**d**). Films were formed on the surfaces of glass (**a**–**c**) and fluoroplast (**d**). The average diameter of the films is approximately 1.5 cm.

Alginate films with and without MSNs were prepared by casting from a solution. At the same time, their structure strongly depended on the nature of the substrate (Figure 5). The films obtained on the glass surface were more uniform. The introduction of MSNs into the alginate matrix led to a change in the color and transparency of the films. It is known that the main way to prevent the dissolution of alginate films in water is to crosslink them with calcium ions. According to known data [22], using aqueous ethanol (10–40 vol%) solutions of CaCl_2_ for this purpose should facilitate the formation of more uniform films. This is exactly what was observed in our experiments (Figure S8). However, the high ethanol content led to the washing out of quercetin from the films. Given this fact, the optimal ethanol concentration was 10 vol%.

**Figure 6:**
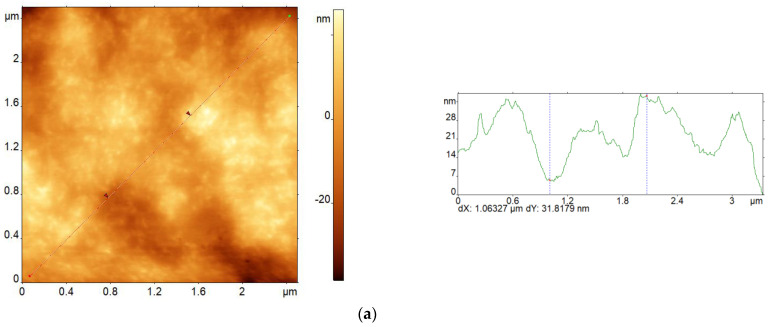

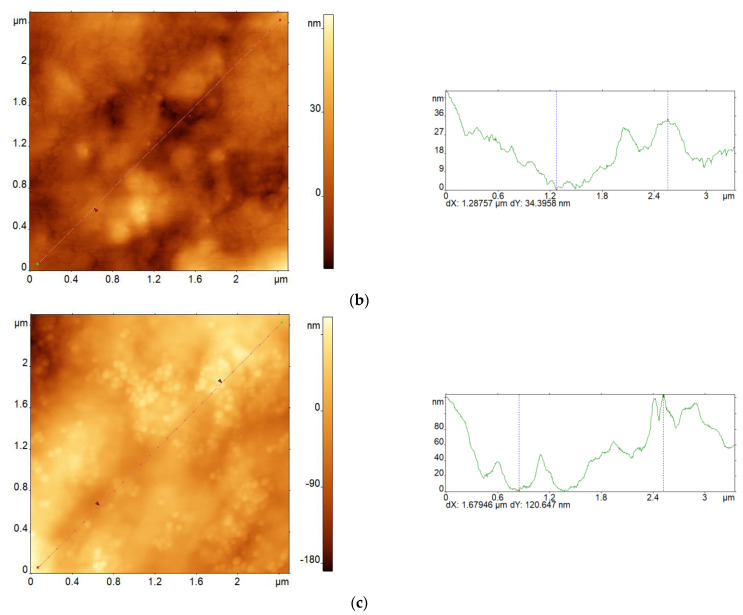
AFM images of sodium alginate films (**a**,**b**) and alginate/MSN composite (**c**) before (**a**,**c**) and after crosslinking with Ca^2+^ ions (**b**), as well as corresponding cross sections of the surface relief.

Figure 6 shows AFM images of pristine alginate films (Figure 6a,b), an alginate/MSN composite (Figure 6c), and corresponding cross sections of their surface relief. Note that the nonuniformity of the surface relief of all the films slightly increased after crosslinking. Thus, the mean roughness (*R*_a_) of the pristine and cross-linked alginate films was equal to 8.0 and 10.4 nm, respectively. This result agrees well enough with the experimental data obtained earlier [22]. The MSNs that were introduced into the alginate matrix partially segregated at the interface with air, which also led to an increase in the nonuniformity of the surface relief (the *R*_a_ value for the pristine composite film was equal to 30.4 nm).

The mechanical stiffness of the films was evaluated by force spectroscopy using an atomic force microscope (see Supporting Information pages 6–8 in [35]). The analysis of this data indicates that the stiffness of all the films increased after crosslinking, since the slope of the corresponding force curves decreases (Figure S9). In addition, the presence of the MSNs in the alginate matrix can also somewhat increase the film stiffness (such a pattern is observed both before and after composite film treatment with CaCl_2_ solution, Figure S10). Note that both the pristine alginate and composite films displayed approximately the same ability to swell: exposure to water for 1 h led to a weight increase of the alginate and composite films of ≈70% and ≈50%, respectively.

Thus, the possibility of creating composites based on a biocompatible polymer of sodium alginate and synthesized MSNs has been demonstrated. Such composites can be of interest in, for example, dressings and dental materials. This is indicated, in particular, by the data of the recently published paper [36] (see also review [37] and the references therein).

### 3.3. Study of the Kinetics of the Encapsulated Drug Release from the MSNs and Alginate/MSN Composites into the Aqueous Medium

The experiments for the study of the kinetics of the encapsulated drug release were carried out under static and quasi-dynamic conditions using MSNs synthesized on a hybrid template in a neutral medium.

**Figure 7:**
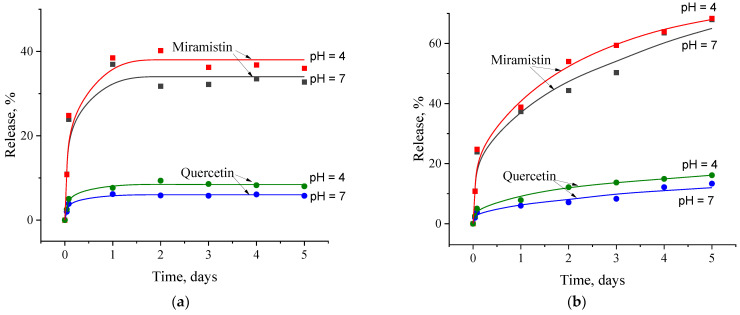
Kinetic curves of the release of miramistin and quercetin from MSNs into neutral and acidic media under static (**a**) and quasi-dynamic (**b**) conditions.

Kinetic curves describing the release of the encapsulated drugs from the MSNs into an aqueous medium with different pH values are shown in Figure 7. It can be seen that the nature of this process in static and quasi-dynamic modes differs markedly. In the first case, the amounts of both released drugs first increased, reached some equilibrium values, and then remained practically unchanged (Figure 7a). The miramistin and quercetin release into the neutral dispersion medium was about 35 wt% and 6 wt%, respectively, of their encapsulated amounts. A decrease in the dispersion medium pH to ≈4 led to increases in the release rate of both drugs and their equilibrium content in the aqueous medium. This result is quite consistent with what we observed earlier for the miramistin + curcumin pair [16].

Under quasi-dynamic conditions, the release of both encapsulated drugs from MSNs was much faster and more complete (see Figure 7b). Thus, approximately 60 wt% and 70 wt% of the encapsulated miramistin were released in the neutral and acidic media, respectively, over 5 days. The same trend was observed for quercetin. Herein, the proportion of this drug that passed into the acidic dispersion medium was 15–16 wt%. It should be emphasized that in both cases considered, the amount of miramistin released into the dispersion medium did not exceed its CMC. This is precisely why the amount of quercetin that desorbed from the MSNs was commensurate with its equilibrium solubility in water. Note that an increase in desorption time over 7 days led to a slight increase in the release rate of this hydrophobic drug. This may be a consequence of the beginning of the hydrolytic destruction of the silica matrix.

Under quasi-dynamic conditions, the amount of miramistin that released from the MSNs correlated linearly with the square root of the time in the interval from 1 to 10 days. (As an example, Figure S11 shows the data for a neutral medium.) This indicates that the release rate of the templating antiseptic from the silica matrix into the dispersion medium is determined by its diffusion through the pores of the MSNs. Note that in this case, the rate of silica matrix “swelling” (i.e., the penetration of water into it) had practically no effect on the process, in contrast to the situation observed in the kinetics study of the miramistin release from the MSNs synthesized in an alkaline medium [38]. This difference is a consequence of the different sizes and porous structures of the MSNs used in [38] and in this work. 

The introduction of MSNs into alginate films quite naturally led to a significant decrease in the release rate of the encapsulated drugs into the surrounding aqueous medium. Thus, about 1.5–2 wt% of miramistin and less than 1 wt% of quercetin was released into the neutral dispersion medium over 10 days. A decrease in pH led to an increase in the proportion of desorbed drugs by about 1.5–2 times. 

Thus, the above data indicate that there is an opportunity to control the release rate of drugs encapsulated in MSNs. Note that the composite we obtained was characterized by the very slow release of the encapsulated drugs, which is necessary for the treatments of a wide range of diseases of the oral cavity. At the same time, the release rates of the drugs can be increased by creating a composite with a porous structure. This opens up the possibility of their use in the creation of new materials for different biomedical purposes.

In conclusion, we will focus on experiments examining the biological activity of the prepared composites.

### 3.4. Study of the Biological Activity of Alginate/MSN Composites

The study of the antibacterial activity of the alginate/MSN composites in vitro was carried out using the agar diffusion method against Gram-positive *Staphylococcus aureus* ATCC 29213. Taking into account the differences in the stiffnesses of the composite films before and after their crosslinking with Ca^2+^ cations as well as their differential abilities to dissolve in a liquid medium, it was of interest to evaluate the antibacterial activity of both types of films. Sodium alginate films lacking MSNs were used as a control. 

**Figure 8:**
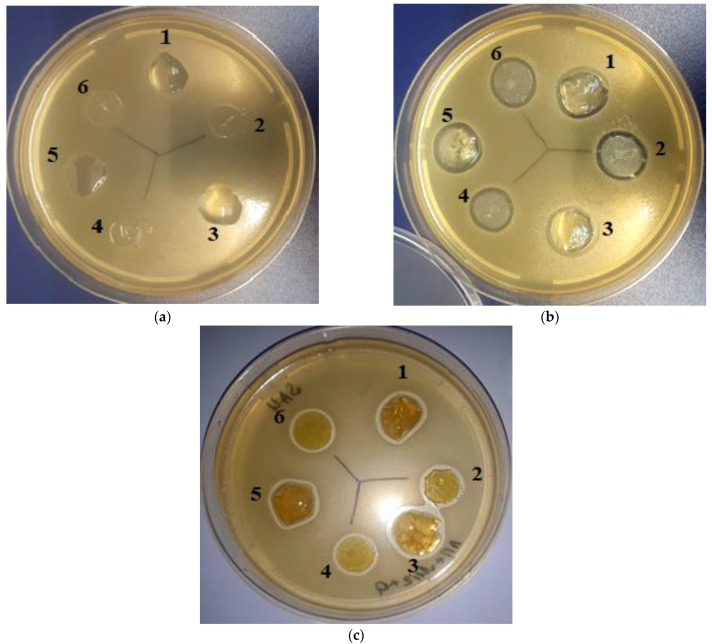
Inhibition of *Staphylococcus aureus* by sodium alginate films (**a**) and alginate/MSN composites containing pure miramistin (**b**) or its combination with quercetin. (**c**) 1, 3, 5—non-crosslinked samples; 2, 4, 6—samples crosslinked with Ca^2+^ ions.

The results of the experiments showed that the alginate/MSN composites exhibited pronounced activity against the *Staphylococcus aureus* Gram-positive culture. This is indicated by the presence of clearly defined zones of bacterial growth inhibition when using both non-crosslinked and crosslinked composite films (Figure 8), even over a very short pre-diffusion time (2 h) with the encapsulated drugs. At the same time, there were no significant differences in the inhibition zone diameters. On the contrary, the control samples did not show any antibacterial activity.

Despite data in the literature on the bactericidal properties of quercetin [31,32], its presence within the MSNs did not lead to any noticeable changes in the inhibition zone diameters (see Figure 8b,c, Table 1). Obviously, in our case, the amount of the drug released from the MSNs was insufficient to provide a significant antibacterial effect. Taking into account the data we obtained earlier [39], it can be expected that this effect would be noticeable with an increase in the pre-diffusion time. In addition, we believe that the antioxidant properties of quercetin should promote wound healing using dressings based on such composites.

## 4. Conclusions

The possibility of creating bifunctional MSNs with a high loading capacity and a controllable release rate has been demonstrated. The MSNs were obtained using a one-pot sol–gel synthesis on a hybrid template, which consisted of micelles of the amphiphilic antiseptic miramistin with the solubilized biologically active compound quercetin. This approach to the synthesis of MSNs and their loading with hydrophobic compounds made it possible to increase (by 40 times) the quercetin solubility in water, which should also contribute to an increase in quercetin’s bioavailability.

The possibility of creating composites based on a biocompatible polymer of sodium alginate and MSNs containing both miramistin and its combination with quercetin has been demonstrated for the first time. The resulting composites exhibited antibacterial activity against a *Staphylococcus aureus* Gram-positive culture. At the same time, the release rate of the drugs from the composite materials was noticeably reduced, potentially providing a prolonged therapeutic effect, which can be very important in the development wound dressings or dental materials.

The proposed approach to the preparation of bifunctional MSNs can be applied to the encapsulation of various hydrophobic and amphiphilic compounds. This opens up opportunities for creating not only multifunctional MSNs but also MSN-based composites characterized by a wide range of biological activity. In this case, other biocompatible polymers can also act as a matrix. The results obtained in this work are of particular interest concerning the solving of biomedical problems related to the delivery and controlled release of several drugs at once.

## Figures and Tables

**Table 1:** The ratio of the diameter of the zone of inhibition of growth of bacteria *Staphylococcus aureus* and composite film diameter.

Alginate/MSN Composite	Non-Crosslinked Films	Crosslinked Films
Drug: miramistin	1.27 ± 0.06	1.26 ± 0.03
Drugs: miramistin, quercetin	1.23 ± 0.04	1.28 ± 0.03

## Data Availability

Data are contained within the article and supplementary materials.

## Supplementary Materials

The following supporting information can be downloaded at: https://www.mdpi.com/article/10.3390/pharmaceutics15122675/s1, Figure S1: Dependences of the optical density of quercetin ethanol solutions and miramistin aqueous solutions on their concentration; Figure S2: Typical absorption spectra of an aqueous solution of miramistin and an ethanol solution of quercetin; Figure S3: Absorption spectra of miramistin micellar solutions with a concentration of 6 mM and 9 mM, recorded 1 h to 6 days after the start of the quercetin solubilization process; Figure S4: Normalized absorption spectra of aqueous micellar solutions of miramistin with solubilized quercetin recorded before and after the addition of an ammonia solution; Figure S5: HRTEM image of MSNs synthesized on miramistin micelles in a neutral medium; Figure S6: FTIR spectra of quercetin, miramistin, and MSNs containing both of these drugs; Figure S7: TGA curves of MSNs synthesized on miramistin micelles with solubilized quercetin in neutral and alkaline media; Figure S8: Photographs of sodium alginate films and alginate/MSNs composites containing pure miramistin or its combination with quercetin; Figure S9: Force curves reflecting the stiffness of pure alginate films before and after crosslinking; Figure S10: Force curves reflecting the stiffness change of pure alginate films in comparison with composite films before and after crosslinking with Ca^2+^ ions; Figure S11: The kinetic curve of miramistin release from MSNs (synthesized on a hybrid template in a neutral medium) into deionized water under quasi-dynamic conditions.
