# Supplementary material for: Antibacterial Bionanocomposites Based on Drug-Templated Bifunctional Mesoporous Silica Nanocontainers
Source: Pharmaceutics. 2023 Nov 25;15(12):2675. doi: 10.3390/pharmaceutics15122675 (PMC10748164; doi:10.3390/pharmaceutics15122675)
Supplement: Supplementary file 1 [file pharmaceutics-15-02675-s001.zip › pharmaceutics-2703772-supplementary.pdf]

Supporting information

(a)

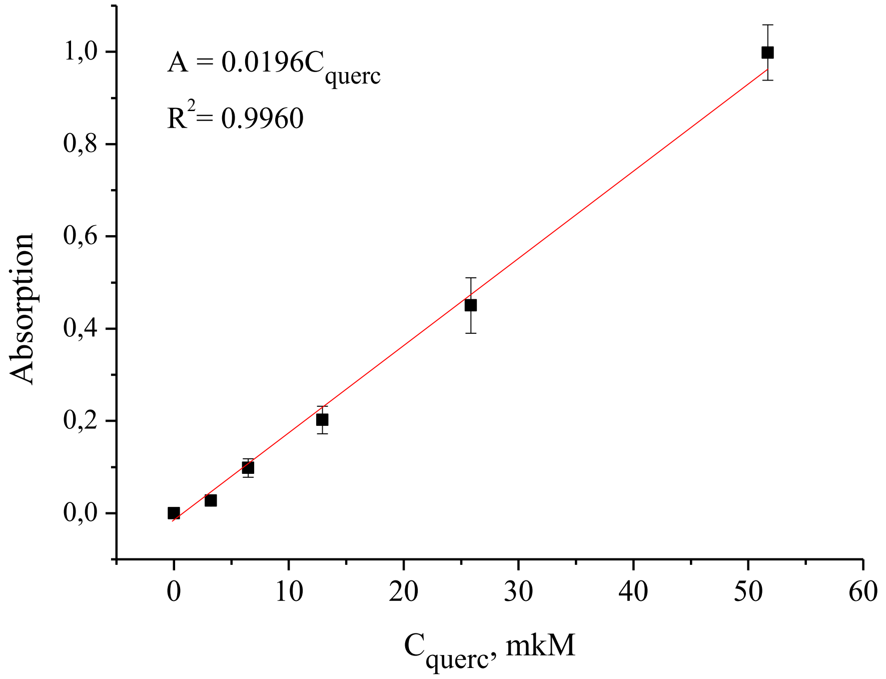

(b)

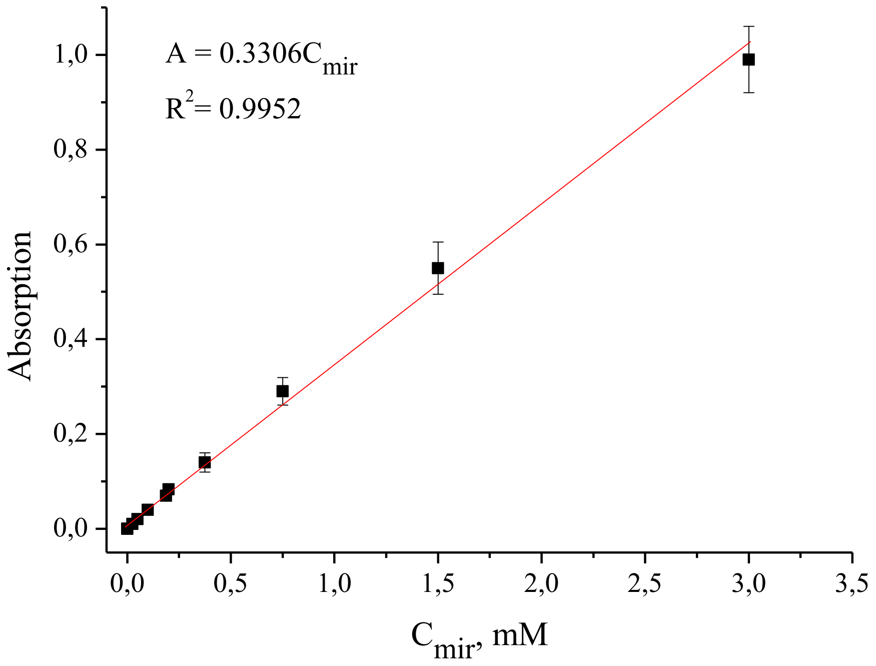

Figure S1. Dependences of the optical density of quercetin ethanol solutions at a wavelength of 375 nm (a) and miramistin aqueous solutions at a wavelength of 263 nm (b) on their concentration.

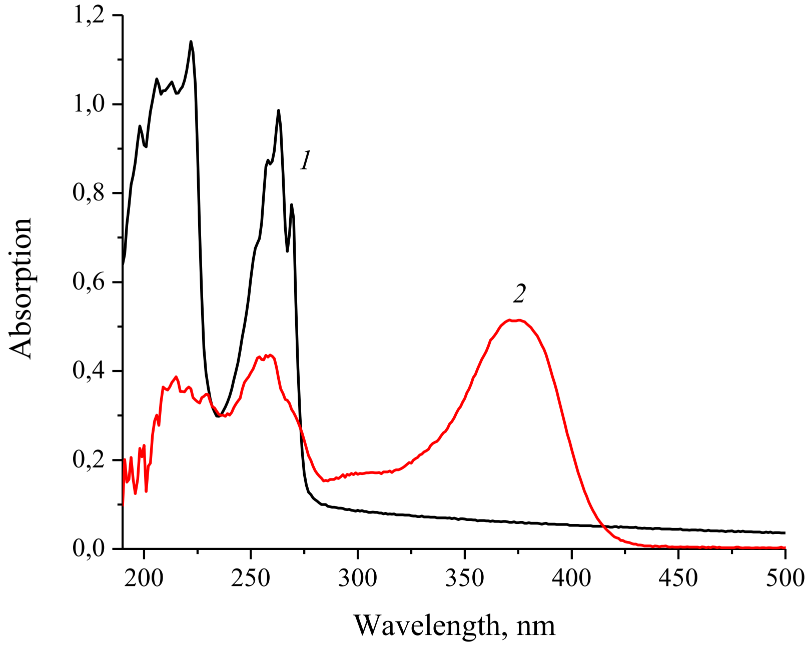

Figure S2. Typical absorption spectra of an aqueous solution of miramistin (*1*) and an ethanol solution of quercetin (*2*).

(a)

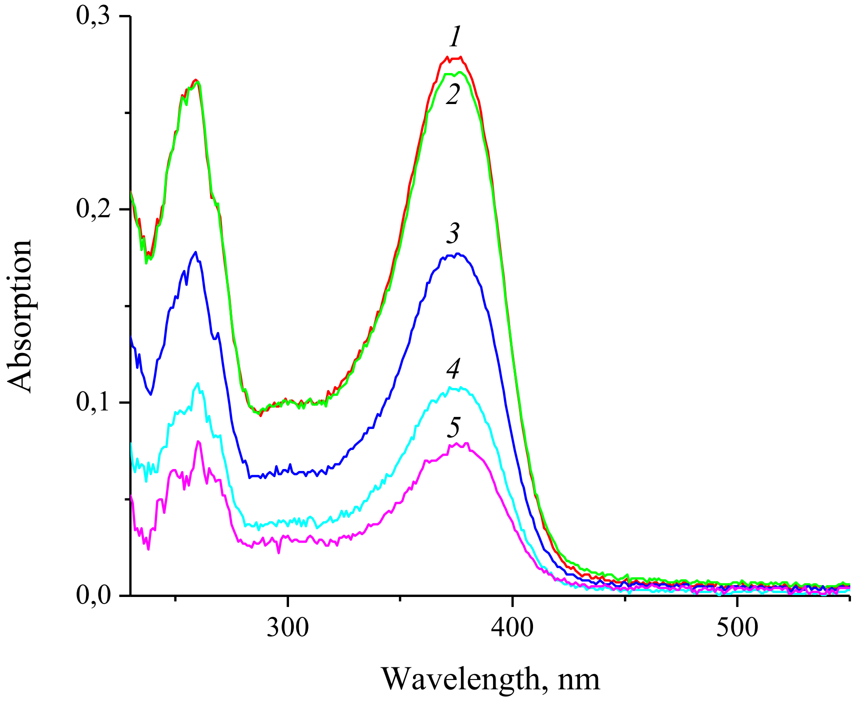

(b)

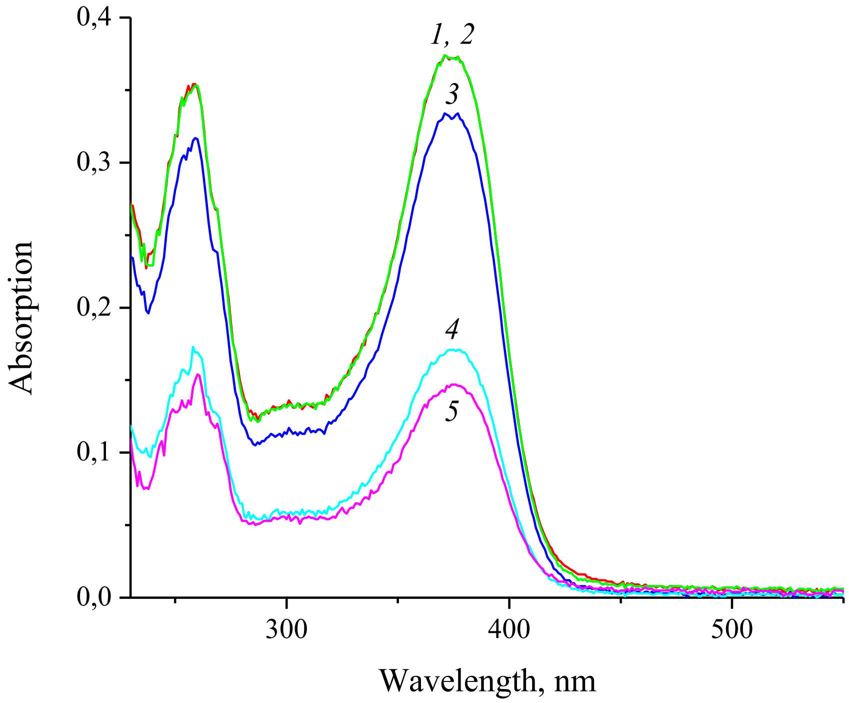

Figure S3. Absorption spectra of miramistin micellar solutions with a concentration of 6 mM (a) and 9 mM (b), recorded after 1 h (*1*), 3 h (*2*), 5 h (*3*), 3 days (*4*), and 6 days (*5*) after the start of the quercetin solubilization process.

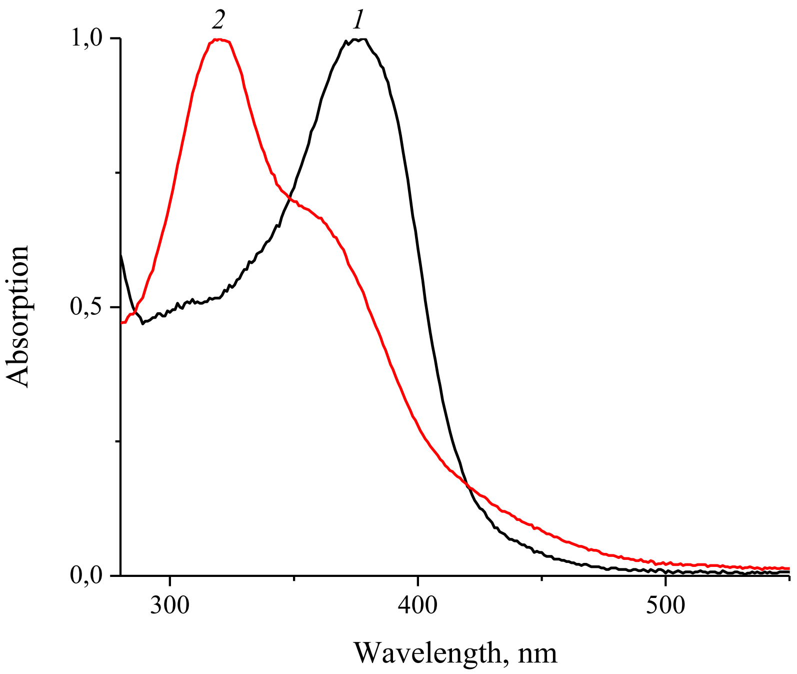

Figure S4. Normalized absorption spectra of aqueous micellar solutions of miramistin with solubilized quercetin recorded before (*1*) and after (*2*) the addition of an ammonia solution.

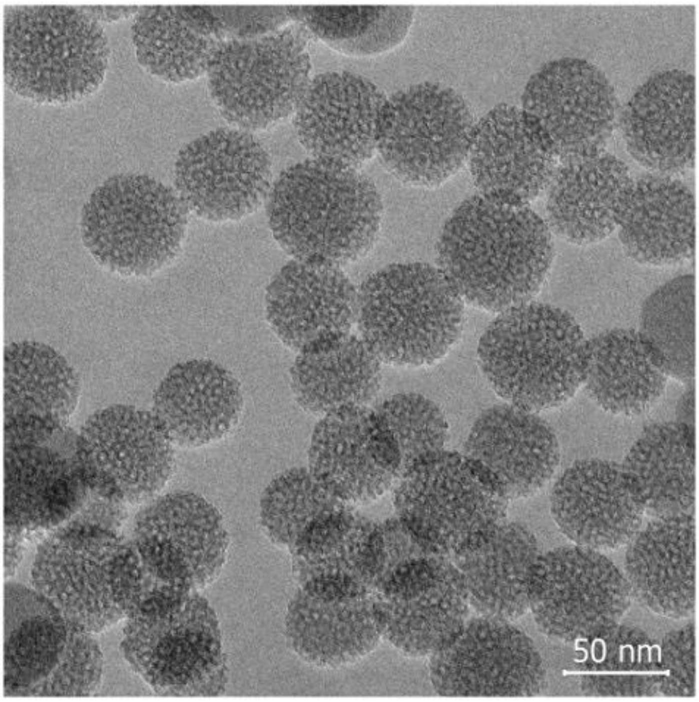

Figure S5. HRTEM image of MSNs synthesized on miramistin micelles in a neutral medium.

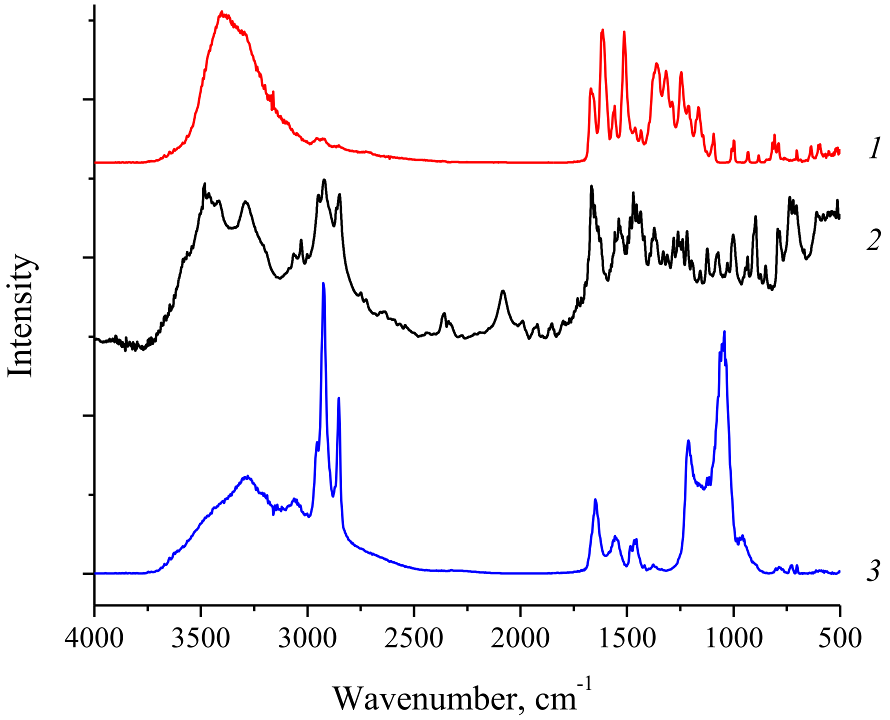

Figure S6. FTIR spectra of quercetin (*1*), miramistin (*2*), and MSNs containing both of these drugs (*3*).

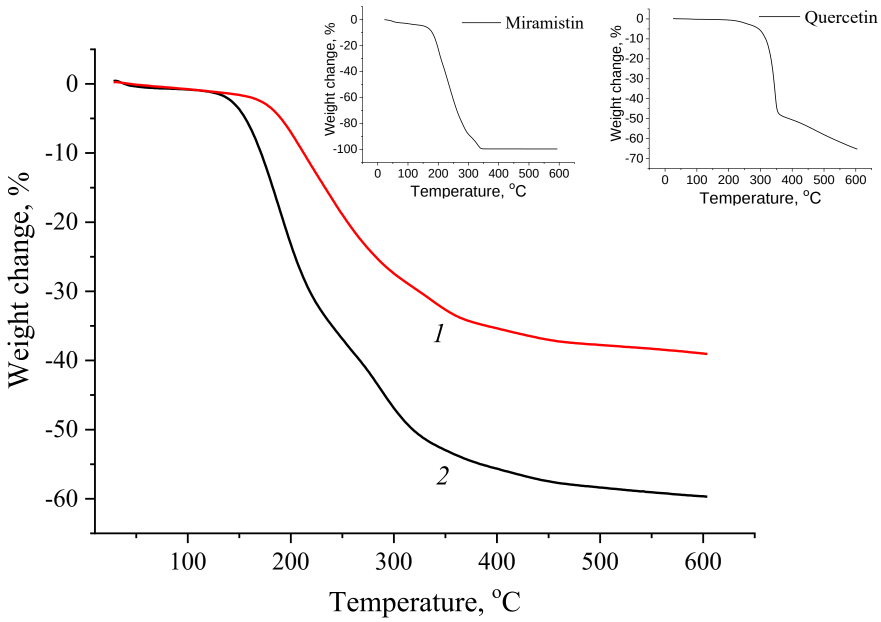

Figure S7. TGA curves of MSNs synthesized on miramistin micelles with solubilized quercetin in a neutral (*1*) and alkaline (2) medium. TGA curves for pure miramistin and quercetin are shown as insets.

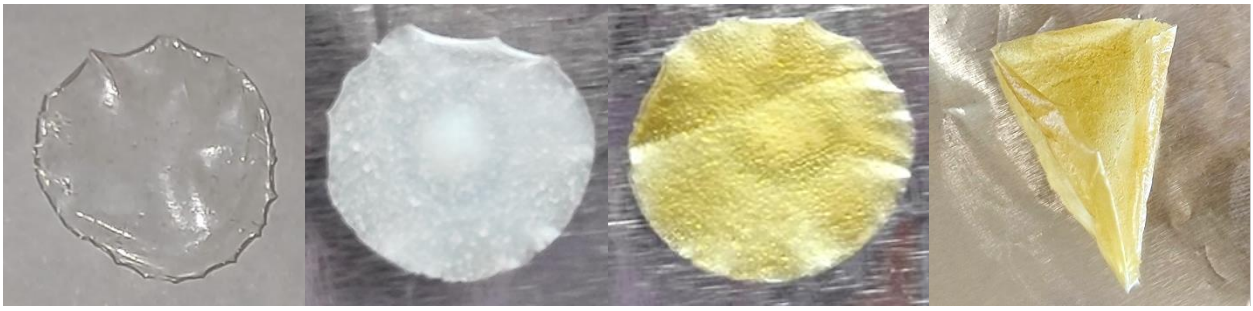

(a) (b) (c) (d)

Figure S8. Photographs of sodium alginate films (a) and alginate/MSNs composites containing pure miramistin (b) or its combination with quercetin (c, d). The films are crosslinked with Ca^{2+} ions in the water–ethanol (10 vol. %) mixture (a, b, c) and in distilled water (d).

The mechanical stiffness of the samples was evaluated by analysis of force curves obtained by force spectroscopy. The force curve represents the dependence of cantilever deflection d measured in nm on the vertical displacement of the piezoscanner z measured in nm. The force of cantilever interaction F with the sample was determined according to Hooke's law:

$$F = kd,$$

where k is the cantilever stiffness equal to 3.5 H/m. The stiffness of the samples was determined by the slope angle of the curve [35]. The more the slope of the experimental curve differs from the slope of the calibration force curve taken on a solid reference substrate, the softer the sample is.

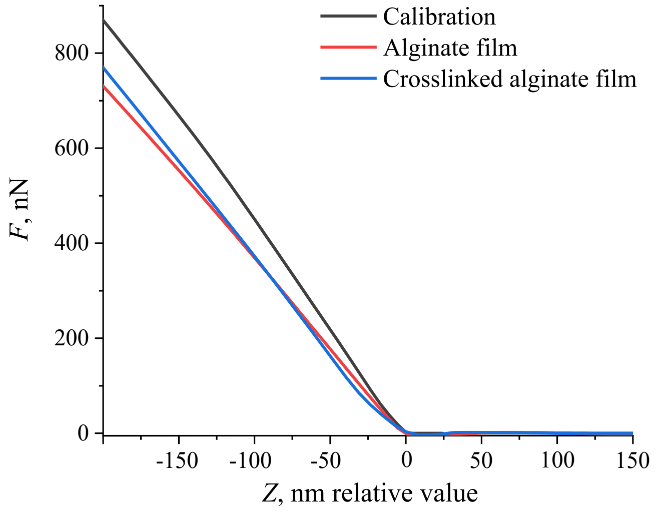

Figure S9. Force curves reflecting the stiffness of pure alginate films before and after crosslinking.

(a)

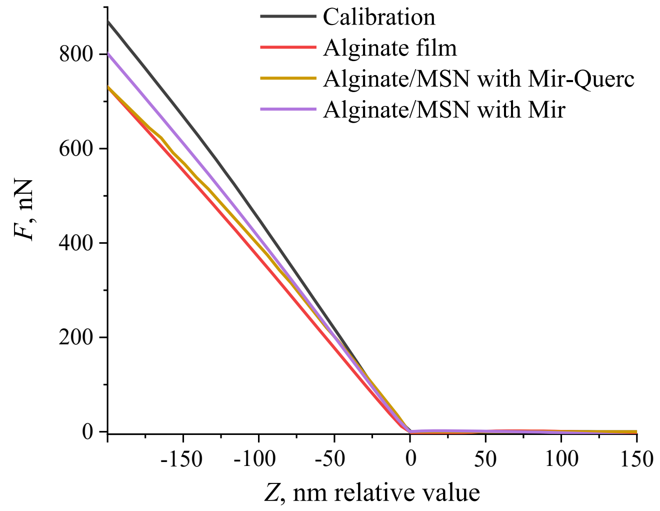

(b)

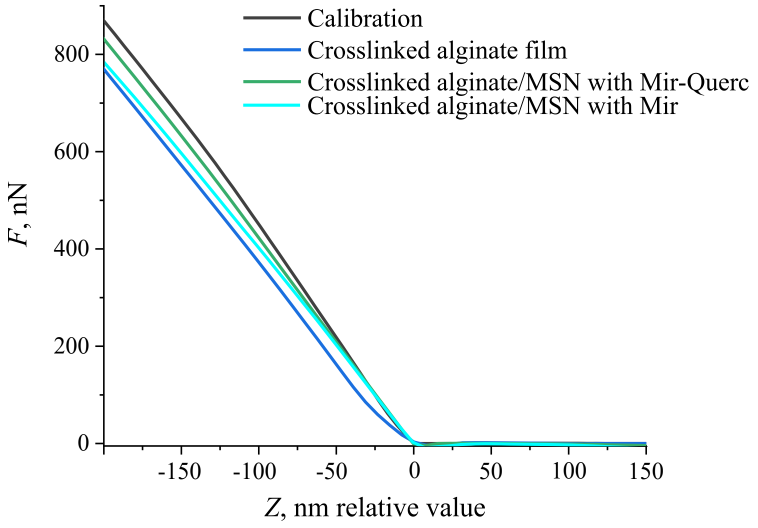

Figure S10. Force curves reflecting the stiffness change of pure alginate films in comparison with composite films before (a) and after (b) crosslinking with Ca^{2+} ions.

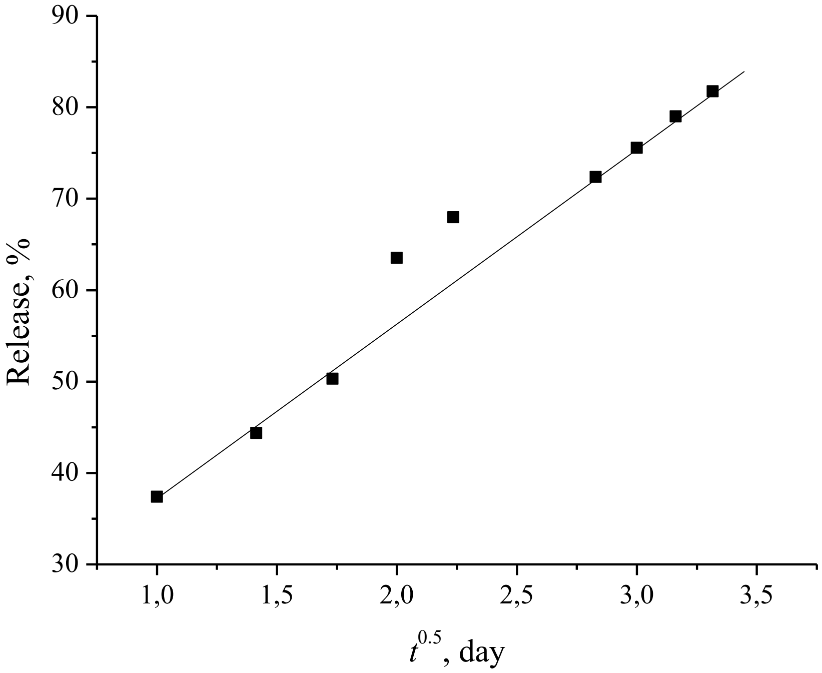

Figure S11. The kinetic curve of the miramistin release from MSNs (synthesized on a hybrid template in a neutral medium) into deionized water in a quasi-dynamic conditions.
